# Comparing Computed Tomography–Derived Augmented Reality Holograms to a Standard Picture Archiving and Communication Systems Viewer for Presurgical Planning: Feasibility Study

**DOI:** 10.2196/18367

**Published:** 2020-09-24

**Authors:** David Dallas-Orr, Yordan Penev, Robert Schultz, Jesse Courtier

**Affiliations:** 1 Department of Bioengineering and Therapeutic Sciences University of California, San Francisco San Francisco, CA United States; 2 Department of Bioengineering University of California, Berkeley Berkeley, CA United States; 3 Department of Radiology Mission Bay Hospital University of California, San Francisco San Francisco, CA United States; 4 Department of Radiology and Biomedical Imaging University of California, San Francisco San Francisco, CA United States

**Keywords:** augmented reality, mixed reality, picture archiving and communication system, presurgical planning, new technology evaluation, medical imaging, surgery

## Abstract

**Background:**

Picture archiving and communication systems (PACS) are ubiquitously used to store, share, and view radiological information for preoperative planning across surgical specialties. Although traditional PACS software has proven reliable in terms of display accuracy and ease of use, it remains limited by its inherent representation of medical imaging in 2 dimensions. Augmented reality (AR) systems present an exciting opportunity to complement traditional PACS capabilities.

**Objective:**

This study aims to evaluate the technical feasibility of using a novel AR platform, with holograms derived from computed tomography (CT) imaging, as a supplement to traditional PACS for presurgical planning in complex surgical procedures.

**Methods:**

Independent readers measured objects of predetermined, anthropomorphically correlated sizes using the circumference and angle tools of standard-of-care PACS software and a newly developed augmented reality presurgical planning system (ARPPS).

**Results:**

Measurements taken with the standard PACS and the ARPPS showed no statistically significant differences. Bland-Altman analysis showed a mean difference of 0.08% (95% CI –4.20% to 4.36%) for measurements taken with PACS versus ARPPS’ circumference tools and –1.84% (95% CI –6.17% to 2.14%) for measurements with the systems’ angle tools. Lin’s concordance correlation coefficients were 1.00 and 0.98 for the circumference and angle measurements, respectively, indicating almost perfect strength of agreement between ARPPS and PACS. Intraclass correlation showed no statistically significant difference between the readers for either measurement tool on each system.

**Conclusions:**

ARPPS can be an effective, accurate, and precise means of 3D visualization and measurement of CT-derived holograms in the presurgical care timeline.

## Introduction

Picture archiving and communication systems (PACS) allow for easy storage and viewing of medical imaging information. Traditional PACS viewers present images in x-ray, computed tomography (CT), and magnetic resonance imaging (MRI) data on a 2-dimensional (2D) workstation screen to be examined by a surgical team in preparation for a complex procedure [1,2]. While these systems have been shown to be accurate and easy to use for the analysis of medical images [3], they are also limited by their requirement of a desktop computer, laptop, or smartphone screen [4]. Dias et al [5] report that 2 of the most common problems of traditional PACS are the mismatch between the 2D viewing screen and the real world and the accompanying lack of flexibility and efficiency of use.

Augmented reality (AR) and virtual reality (VR) technologies have the potential to address these shortcomings. AR and VR alike allow for the realistic and interactive digital representation of objects in a 3D space. As such, both technologies are already successfully deployed across a diverse set of applications, including terrestrial navigation [6], architectural modeling [7], automotive engineering [8], and education [9]. The same properties could be applied to present a realistic overlay of medical devices and tools on patients’ anatomy in 3D space on a portable, shared visualization method.

Whereas VR presents an entirely digital representation of objects and their environment, AR allows for the overlay of digital holograms on a live real-world scene. In addition, many VR systems require a dedicated physical play space to allow for the experience of the completely immersive digital experience [10]. These characteristics make AR a more likely candidate for the development of interactive tools assisting the dynamic clinical workflow.

The potential of AR systems to assist in clinical tasks has been extensively reviewed by Uppot et al [11]. Possible use cases include supplementing radiology training; communicating with colleagues, referring clinicians, and patients; and aiding in interventional radiology procedures. Additional uses for AR in medicine include providing simulations for advanced life support training [12], visualizing patient anatomy including tumors [13], and guiding assistants during robotic surgery [14]. The increased spatial understanding of anatomy with AR has been shown to positively impact surgical care during laparoscopic surgery for visualizing hidden patient anatomy [15], resection of neurological tumors without causing new neurological deficit [16], and breast tumor resection by maximizing breast conservation [17]. Multiple other non–patient outcome benefits have been proposed, including overall operating room efficiency [18,19], and more specifically—reduced operating room time, increased surgical precision, and reduced radiation exposure [20].

In order to create an AR model suitable for presurgical planning, the medical image from a CT or MRI scan must first be segmented using a DICOM viewer to visualize only the object or organ of interest. The resulting image is passed onto an image processing software that renders the object’s volumes and surfaces into a 3D scalar field model. This model can later be loaded in a dedicated AR software designed for projecting the image onto an AR or mixed reality headset display. Similar technologies have evaluated the use of AR systems for the visualization of MRI data [21]. However, the focus of this study is the validation of CT-derived holograms. Although the visualization of CT-derived holograms has been assessed, measurement systems for these CT-derived holograms are rarely evaluated or utilized.

As AR becomes more widely used in presurgical planning, it is crucial to know that these systems meet the gold standard for medical image measurement. This study aims to validate the feasibility, safety, and efficacy of a novel ARPPS, compared to a standard-of-care PACS viewer, in order to support its use in the presurgical visualization and measurement of CT-derived imaging of patient anatomy and surgical tools.

## Methods

### Materials

**Figure 1:**
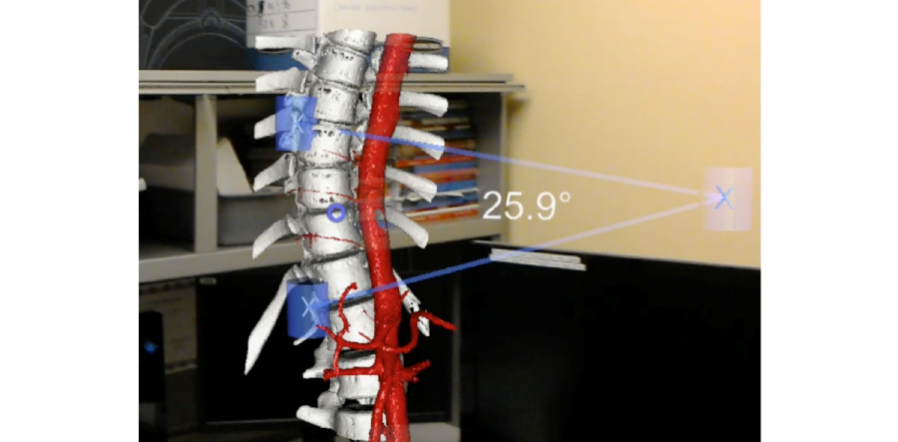
The RadHA ARPPS version 3.3 displaying a spine model with a vascular model overlay and an angle measurement of thoracic kyphosis.

A CT image data set was generated using Discovery CT750 HD (GE Healthcare). The object imaged was a CT dose meter phantom (model 137856101, GE Healthcare) compliant with the American College of Radiology standards. The PACS used for standard-of-care comparison was Osirix MD version 10.0 (Pixmeo SARL; FDA 510(k) K101342) [22]. The experimental PACS was the RadHA ARPPS version 3.3 (University of California, San Francisco) (Figure 1), as viewed on HoloLens generation 1 headset (Microsoft Corp). A MT-912 Digital Light Meter (Urceri) was used to measure the background light intensity.

### Procedure

**Figure 2:**
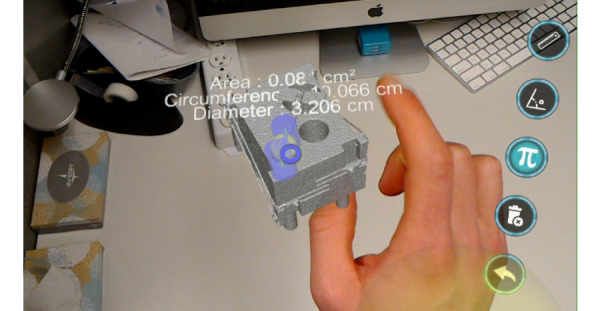
The RadHA ARPPS version 3.3 displaying a computed tomography (CT)-derived 3D hologram of a CT dose meter phantom with diameter and circumference measurements and selectable icons.

**Figure 3:**
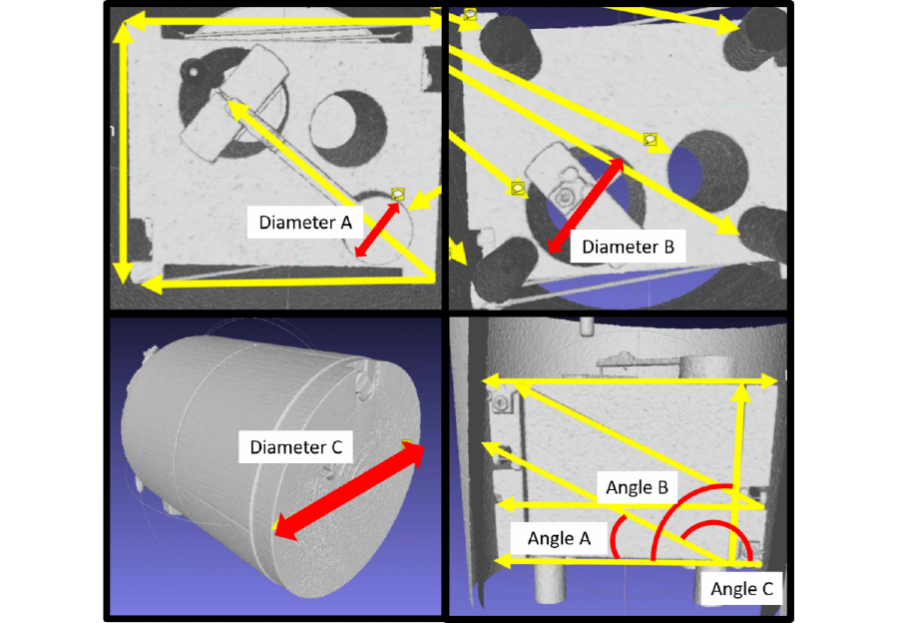
Computed tomography (CT) dose meter phantom diameters and angles as per the manufacturer's specifications.

The CT dose meter phantom DICOM (digital imaging and communications in medicine) file was converted to an OBJ file (object file, Wavefront Technologies) and uploaded to the ARPPS for viewing on the HoloLens. The circumference and angle measurement tools of both the standard PACS and the ARPPS were used to measure diameters (Figure 2) and angles, respectively, with reference to the manufacturer-specified parameters of the CT dose meter phantom (Figure 3).

A range of low, medium, and high clinical measurements were selected for anthropomorphic correlation of the phantom’s diameter and angle parameters (Table 1). Two readers measured each of the phantom parameters 10 times independently of each other starting with the ARPPS. The readers were blinded to the manufacturer-provided measurements. Testing was completed in an office with a background light intensity of 152.1 lux.

**Table 1:** Clinical significance of the CT dose meter phantom measurements.

Object	Manufacturer-specified size	Clinical guideline
Diameter A	3.215 cm	Mitral valve repair valve sizing [23] (mitral annulus diameter 3.15 cm)
Diameter B	5.0 cm	Elective abdominal aortic aneurysm repair in women [24] (5.0-5.4 cm)
Diameter C	21.31 cm	Pediatric abdominal diameter
Angle A	26.57°	Scoliosis evaluation [25] (bracing Cobb angle 29-40°)
Angle B	90.0°	Proximal tibial alignment [26] (normal lateral distal tibial angle 90°)
Angle C	153.43°	Pediatric hip evaluation [27] (normal pediatric femoral shaft angle 160°)

### Statistical Analysis

All statistical analyses were performed using Microsoft Excel version 1903. The interrater reliability of the readers was verified using Lin’s concordance correlation coefficient for both the circumference and angle tools [28]. Shapiro-Wilk test was performed to verify the normality of the differences of each set of measurements in order to satisfy the requirements of performing a nonparametric method of analysis such as a Bland-Altman analysis [29]. Bland-Altman analysis was used to evaluate the agreement between measurements taken with the standard PACS and the ARPPS.

## Results

**Figure 4:**
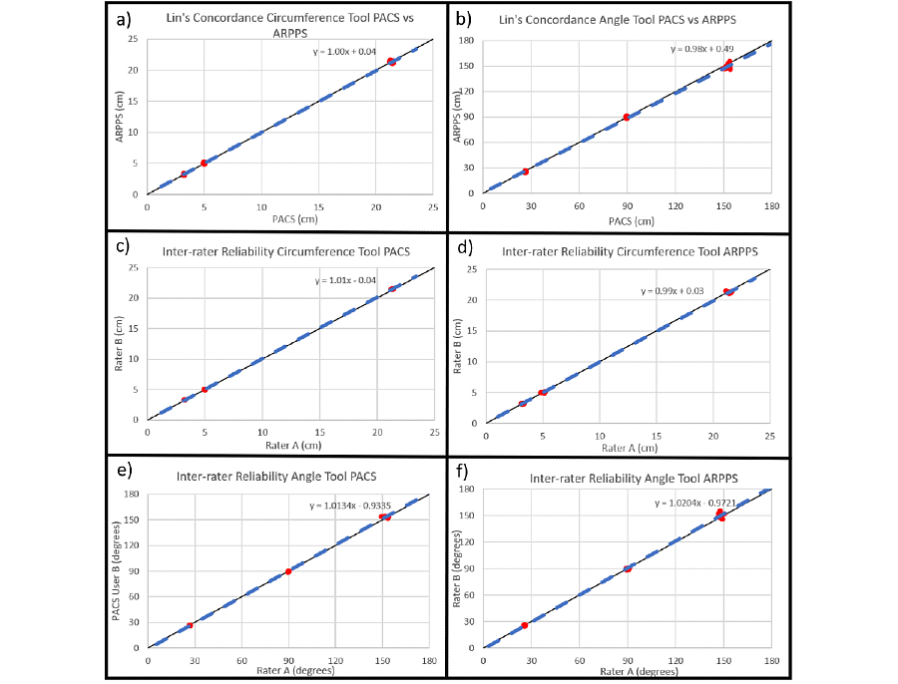
Lin’s concordance plots of a) circumference tool, b) angle tool; interrater reliability plots of c) circumference tool for the picture archiving and communication system (PACS), d) circumference tool for augmented reality presurgical planning system (ARPPS), e) angle tool for PACS, f) angle tool for ARPPS.

Lin’s concordance correlation coefficient showed almost perfect concordance of the standard PACS viewer and the ARPPS (Figure 4, Table 2). Additionally, no significant difference in interrater reliability was observed for the circumference and angle tool measurements for both the PACS and ARPPS separately (Figure 4, Table 2).

**Figure 5:**
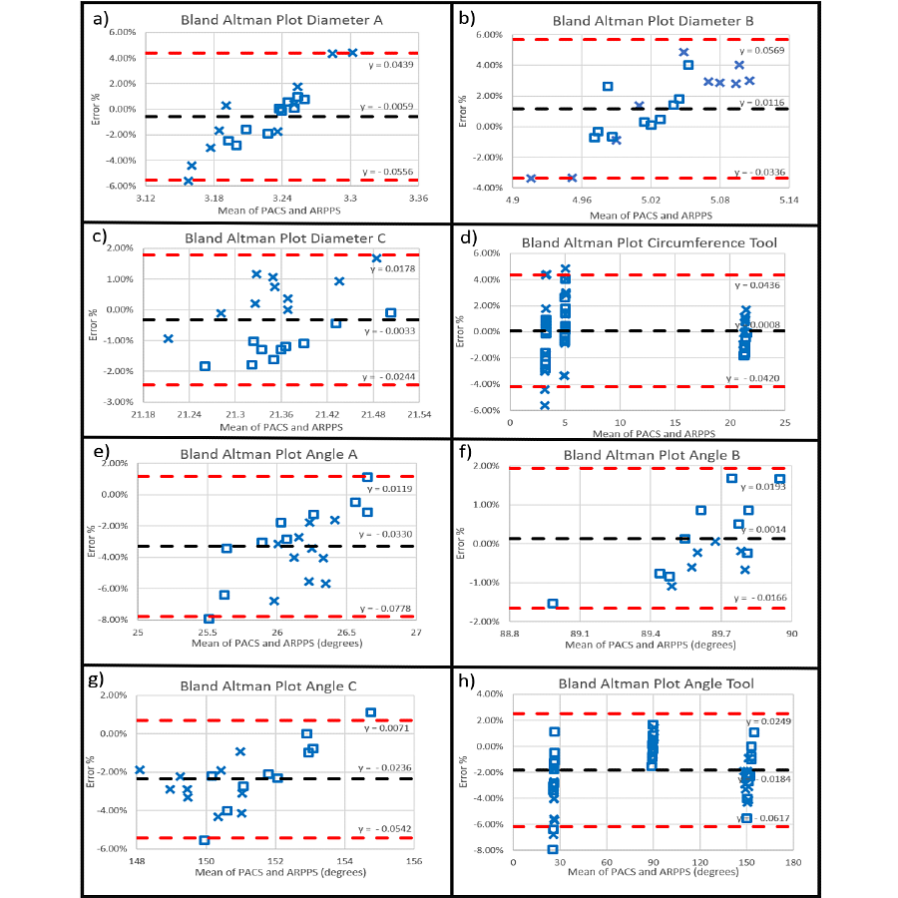
Bland-Altman plots for the circumference tool measurements for a) diameter A, b) diameter B, c) diameter C, d) all diameters combined, and of the angle tool measurements for e) angle A, f) angle B, g) angle C, h) all angles combined.

The Shapiro-Wilk tests failed to reject the null hypothesis of normality (Table 3). Bland-Altman plots evaluating the circumference tool showed an average bias of 0.08% with a 95% CI –4.20% to 4.36%. Bland-Altman plots evaluating the angle tool showed an average bias of –1.84% with a 95% CI –6.17% to 2.14%. The bias and confidence intervals of each of the 3 measures for the circumference and angle tools are reported in Table 3. The Bland-Altman plots of each of the measurements, as well as the combined measurements are shown for the circumference tool (Figure 5 a-d) and angle tool (Figure 5 e-h).

**Figure 6:**
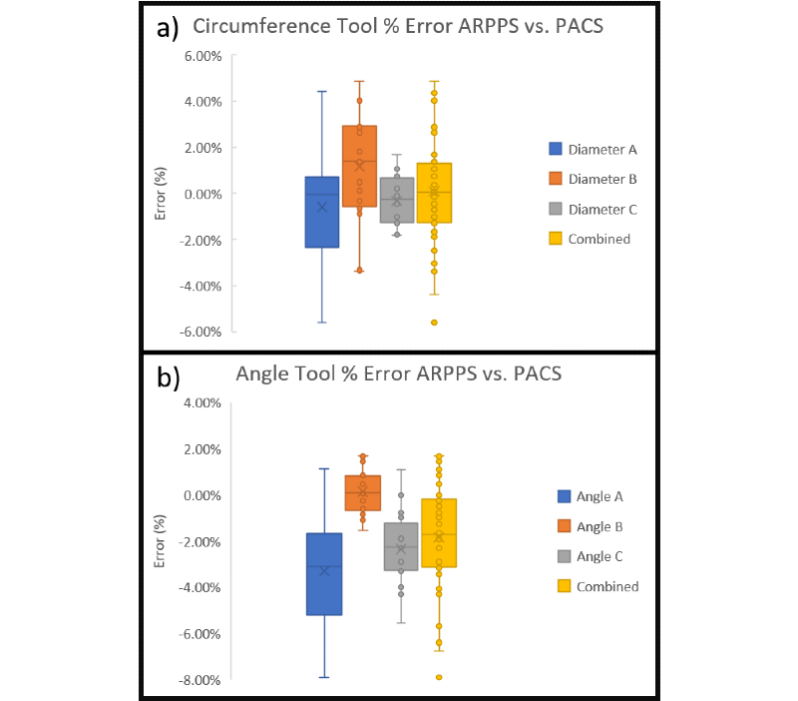
Whisker plot comparisons of percent error of the augmented reality presurgical planning system (ARPPS) versus the standard picture archiving and communication system (PACS) for a) circumference tool, b) angle tool.

The variability of the percent error of each of the measurements using the ARPPS as compared to using the standard PACS are visualized in individual box plots in Figure 6.

**Table 2:** Lin's concordance correlation coefficients and interrater reliability.

Tools	Concordance correlation coefficient	Interrater reliability PACS^a^ standard DICOM^b^ viewer	Interrater reliability ARPPS^c^
Circumference tool	1.00	1.01	0.99
Angle tool	0.98	1.01	1.02

^a^PACS: picture archiving and communication system.

^b^DICOM: digital imaging and communications in medicine.

^c^ARPPS: augmented reality presurgical planning system.

**Table 3:** Shapiro-Wilk test for normality of differences and Bland-Altman analysis.

Tools and measurements		Shapiro-Wilk test *P* value	% Bias	Lower limits of agreement, %	Upper limits of agreement, %
**Circumference tool**					
	Diameter A	.5607	–0.59	–5.56	4.39
	Diameter B	.4528	1.16	–3.36	5.69
	Diameter C	.3325	–0.33	–2.44	1.78
	Combined	N/A	0.08	–4.20	4.36
**Angle tool**					
	Angle A	.8304	–3.30	–7.78	1.19
	Angle B	.9685	0.14	–1.66	1.93
	Angle C	.7211	–2.36	–5.42	0.71
	Combined	N/A	–1.84	–6.17	2.49

## Discussion

### Principal Results and Comparison to Prior Work

Both the circumference and angle measuring tools of the ARPPS had an accuracy that was not significantly different as compared to the PACS measurements used in traditional preoperative settings. The circumference tool had an overall bias of 0.08%, which is more accurate than the 0.3% previously reported for a comparable AR system [30]. Similarly, the angle tool had an overall bias of –1.84%, which is more accurate than that previously reported for another 3D reconstruction software already on the market [31].

Interestingly, a decrease in percent error in either circumference or angle tool measurements was associated with an increase in the size of the object and ray length, respectively (Figure 6). This was consistent with a corresponding increase in the ease of manipulation of the hologram for larger objects as reported by both readers. AR and mixed reality–viewing hardware with higher resolution and responsiveness is likely to significantly improve the usability of such systems.

### Limitations

Manipulating objects on the HoloLens can be technically challenging and contain a systematic error. Both readers reported difficulties in determining a clear vertex for angles A and C. However, angle B, which had no reported difficulties in measurement, showed a bias of only 0.14%. In addition, readers reported significant improvements in hologram manipulation dexterity with experience.

### Conclusions

ARPPS can be an effective, precise, and accurate tool for the realistic visualization, manipulation, and measurement of clinically significant angles and circumferences in 3D space. ARPPS measurements are of substantially equivalent accuracy and precision as compared to standard-of-care PACS, similar systems that have previously been awarded the Food and Drug Administration (FDA) clearance as class II medical devices for presurgical planning, and other systems with published data [30,31]. Nonetheless, technological difficulties remain a major barrier to the adoption of such technologies in medical and surgical care settings. To realize the full potential of AR and similar technologies, it is important that the medical community works in concert with device manufacturers to ensure the devices’ real-world feasibility, usability, safety, and efficacy.

## Abbreviations

2D: 2-dimensional
AR: augmented reality
ARPPS: augmented reality presurgical planning system
CT: computed tomography
DICOM: digital imaging and communications in medicine
FDA: Food and Drug Administration
MRI: magnetic resonance imaging
PACS: picture archiving and communication system
VR: virtual reality
